# BARRIERS TO GEROSCIENCE IN CLINICAL COMMUNITY

**DOI:** 10.1093/geroni/igae098.1340

**Published:** 2024-12-31

**Authors:** George Taffet

**Affiliations:** Houston Methodist Hospital, Houston, Texas, United States

## Abstract

I hope geriatricians will lead the application of GeroScience concepts to the care of older patients, and gerotherapeutics will reinvigorate the field of geriatrics. Yet geriatricians have been skeptical in embracing the potential it has to change their lives and that of their patients. A consensus of leaders stated “geriatricians specialize in the care of people with multiple chronic medical conditions that cause challenges with their day-to-day physical and mental functioning.” GeroScience addresses the aging process underpinning most of those medical conditions so the reluctance seems inexplicable. I will review some causes of this “disconnect” and potential strategies to make GeroScience and Gerotherapeutics more accessible and vital to geriatricians. Geroscience papers are infrequently in sources that Geriatricians read and studies don’t choose outcomes relevant to Geriatricians. The outcomes need to be clinically translatable and meaningful. Geriatricians: often disassociate blood results from the patient in front of them thus studies that only show serum studies are undervalued; see heterogeneity all the time and undervalue changes in means; don’t often see the clinical thumbprint in the study creation; frequently think “the horse is out of the barn” for their patients; have little exposure to geroscience during training and don’t know how to fit it into their practices; worry about ineffective “cures to aging” and increasing disparities. The expansion of these points (and others) and the strategies to counter them will be explored.

